# SPATIAL DISTRIBUTION OF NEWBORNS WITH SICKLE CELL TRAIT IN SERGIPE, BRAZIL

**DOI:** 10.1590/1984-0462/2020/38/2018229

**Published:** 2020-03-09

**Authors:** Débora Cristina Fontes Leite, Rosana Cipolotti, Ricardo Queiroz Gurgel, Paulo Ricardo Saquete Martins, Gabriel Dantas Lopes

**Affiliations:** a Universidade Federal de Sergipe, Aracaju, SE, Brazil.; b Universidade Tiradentes, Aracaju, SE, Brazil.

**Keywords:** Sickle cell disease, Sickle cell trait, Neonatal screening, Residence characteristics, Doença falciforme, Traço falciforme, Triagem neonatal, Distribuição espacial

## Abstract

**Objective:**

To use the spatial distribution of the sickle cell trait (SCT) to analyze the frequency of hemoglobin S (HbS) carriers in Sergipe.

**Methods:**

The sample consisted of all individuals born in Sergipe from October 2011 to October 2012 who underwent neonatal screening in the public health system. Tests were carried out in basic health units and forwarded to the University Hospital laboratory, where they were analyzed. We used spatial autocorrelation (Moran’s index) to assess the spatial distribution of heterozygous individuals with hemoglobinopathies.

**Results:**

Among 32,906 newborns, 1,202 showed other types of hemoglobin besides Hemoglobin A. We found a positive correlation between the percentage of black and multiracial people and the incidence of SCT. Most SCT cases occurred in the cities of Aracaju (n=273; 22.7%), Nossa Senhora do Socorro (n=102; 8.4%), São Cristóvão (n=58; 4.8%), Itabaiana (n=39; 4.2%), Lagarto (n=37; 4.01%), and Estância (n=46; 4.9%).

**Conclusions:**

The spatial distribution analysis identified regions in the state with a high frequency of HbS carriers. This information is important health care planning. This method can be applied to detect other places that need health units to guide and care for sickle cell disease patients and their families.

## RESUMO

**Objetivo:**

Basear-se na distribuição espacial do traço falciforme (TF) para analisar a frequência dos portadores da hemoglobina S (HbS) em Sergipe.

**Métodos:**

A amostra foi constituída por todos os indivíduos nascidos em Sergipe, no período de outubro de 2011 a outubro de 2012, submetidos à triagem neonatal pelo Sistema Único de Saúde, ano de início da triagem universal no Estado. Os testes foram realizados em unidades básicas de saúde e encaminhados para o laboratório do Hospital Universitário, onde foram analisados. A análise da distribuição espacial dos indivíduos heterozigotos para hemoglobinopatias foi realizada por autocorrelação espacial (índice de Moran).

**Resultados:**

Dentre os 32.906 recém-nascidos estudados, 1.202 apresentaram outras hemoglobinas além da Hemoglobina A. Houve correlação positiva entre a porcentagem de negros e mestiços e a incidência de TF. A maioria dos casos foi encontrada nos municípios de Aracaju (n=273; 22,7%), Nossa Senhora do Socorro (n=102; 8,4%), São Cristóvão (n=58; 4,8%), Itabaiana (n=39; 4,2%), Lagarto (n=37; 4,01%) e Estância (n=46; 4,9%).

**Conclusões:**

Na análise de distribuição espacial por autocorrelação, identificaram-se regiões no Estado com maior frequência de HbS, o que é de extrema importância para o planejamento do sistema de saúde, podendo a mesma metodologia ser aplicada para identificação de outros locais com maior necessidade de centros para cuidados e orientações a portadores de doença falciforme e seus familiares.

## INTRODUCTION

Sickle cell anemia (SCA) is the most common monogenic disease in the world and is associated with mutant hemoglobin (HbS), which forms polymers in the red blood cells of patients, leading to chronic anemia. SCA is endemic in many regions where malaria is or was prevalent due to the protective nature of the carrier state^1^ and where the proportion of African descendants is higher.^2^ Throughout the American Continent, the distribution of HbS - more prevalent in populations along the eastern coast - closely matches the distribution of people of African descent.^3^

In Brazil, SCA is more prevalent in the Northeast and three states of the Southeast region - São Paulo, Rio de Janeiro, and Minas Gerais. Overall, in the state of Bahia, in the Northeastern region, the incidence was 1:677 live births. The high prevalence of sickle cell disease (SCD) in those regions can be historically explained by the forced migration of individuals brought to Brazil as slaves from Africa during the colonial period, mainly for working in sugar cane plantations in the Northeast and gold mines in the Southeast.^4^

Sickle cell trait (SCT) is a carrier state of SCA with one copy of normal beta globulin gene and one copy of sickle variant gene-producing heterozygous (HbAS).^5^ Although SCT is considered a harmless condition, complications including hypercoagulability, venous thromboembolic events, renal disease, exertional rhabdomyolysis, and exercise-related sudden death have been reported.^6^ Individuals with SCT are often not informed or fully educated about their SCT carrier status, which leads to confusion about health risks and mistrust of the underlying intentions for screening.^7^ Therefore, genetic counseling and guidance are important for families that have a child with HbAS.^8^ The Southeastern region of Brazil has shown a high prevalence of SCT - one SCT carrier in every 27 births. Nevertheless, according to the Ministry of Health, SCT is present in approximately 5.3% of the population in Bahia - the state with the highest percentage.^2^

Patients with SCT are everywhere in Northeastern Brazil. In the state of Sergipe, the prevalence of HbAS among blood donors is 4.1%.^9^ However, the prevalence among newborn infants in the general population, as well as their spatial distribution, is not known. The Newborn Screening Program (NSP) is a public health project that screens all babies for a range of conditions, including phenylketonuria, congenital hypothyroidism, SCD, and cystic fibrosis.^10^ The use of geotechnologies and spatial analysis of HbAS SCT may enable effective health care planning to address the needs of this population.

This study aimed to describe the spatial distribution of individuals with HbAS SCT by using data from the NSP for hemoglobinopathies in the state of Sergipe, Northeastern Brazil.

## METHOD

This study was conducted in the state of Sergipe, the smallest federal unit in terms of territory extension in Brazil (21,910 km^2^). Sergipe is in the Northeastern region and comprises 75 cities, grouped into three mesoregions: Eastern, *Agreste*, and *Sertão Sergipano*. Its Human Development Index (HDI) is 0.681, life expectancy at birth is 72.1 years, and the infant mortality rate is 18 per 1,000 live births.^11^ Approximately 50% of the population lives below the Poverty Index.^12^ The population has many ethnic and national origins, including especially Portuguese, Germans, Italians,^13^ and black Africans.^14^

The studied population included live births in the first year of implementation of NSP for hemoglobinopathies in Sergipe (October 2011 to October 2012), consisting of about 80% of the live births in the state during this period. The remaining 20% of newborn babies were screened at private outpatient clinics and data from this population is not available. The screening program collected heel prick samples up to 30 days after birth, and babies with positive screening were retested. Tests were carried out in basic health units and forwarded to the University Hospital laboratory, where they were analyzed. Isoelectric Focusing Electrophoresis was performed to identify HbAS SCT, as recommended by the Brazilian government directive MS 822/01. Data regarding gender, ethnicity, birth date, and zip code of birth were also collected. Information about population estimates and self-reported ethnicity were gathered from the Brazilian Institute of Geography and Statistics,^15^ available on the website of the Technology Department of the public health system (Departamento de Informática do Sistema Único de Saúde - DATASUS).^16^

We calculated the cumulative incidence of HbAS SCT with the proportion of new cases during the period of study divided by the total population at risk. The existence of spatial patterns of HbAS SCT in Sergipe was measured by the Moran’s Index (I). Analyses were made by area (cities).

We adopted the global spatial autocorrelation Moran’s I statistics to assess the degree of similarity between a certain location and its neighboring units. Positive values (between 0 and +1) were associated with spatial clustering patterns, whereas negative values (between 0 and -1) indicated a spatial dispersion pattern. Moran’s I value close to zero represented a random pattern of distribution. We elaborated a Moran scatter plot to visualize the results. Observations in the lower left (Low-low) and upper right (High-high) quadrants represented potential spatial clusters, while observations in the upper left (Low-high) and lower right (High-low) suggested potential spatial outliers. The slope of the scatter plot corresponded to the value for global Moran’s I.^17^ The location of clusters or hotspots of HbAS SCT were examined by the Local Indicators of Spatial Association (LISA) cluster map. We used the Benjamini-Hochberg False Discovery Rate (FDR) to adjust the p values. We also adopted the Kernel method, a statistical non-parametric interpolation technique, in which a distribution of points or events is transformed into a “continuous risk surface”. This procedure allowed us to filter the variability of the data set, without, however, changing its local characteristics in an essential way.^18^ The analyses were performed with the R-language (R 2.8.1) and TerraView (4.2.2).^19^ We considered significant a p<0.05.

The local Research Ethics Committee approved this study under the Approval Protocol CAAE-06347012.0.0000.0058.

## RESULTS

**Figure 1:**
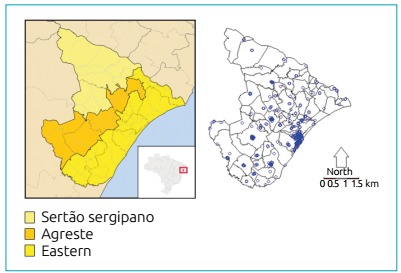
Distribution of HbAS sickle cell trait cases in the cities of Sergipe, 2011-2012.

The study included 32,906 children born in the state of Sergipe from October 2011 to October 2012. A total of 921 children had HbAS SCT, 242 had hemoglobin A (HbA) with another non-S hemoglobin, and 21 were diagnosed with SCD (Table 1). Figure 1 shows the spatial distribution of 921 cases of HbAS Sickle Cell Trait in Sergipe State.

**Table 1:** Distribution of abnormal results identified by neonatal screening for hemoglobinopathies in Sergipe, 2011-2012.

Result	Frequency	% of cases	*Incidence coefficient
AFS	921	76.6%	2.70
AFC	234	19.4%	0.70
AFA2	18	1.5%	0.05
FS	16	1.3%	0.04
AFD	7	0.5%	0.02
FSC	4	0.3%	0.01
FC	1	0.0%	0.003
AF indeterminate variant	1	0.0%	0.003
Total	1,202	100%	-

AFS: heterozygous for Hg S and A; AFC: heterozygous for Hg C and A; AFA2: thalassemia; FS: homozygous for Hg S; AFD: heterozygous for Hg A and D; FSC: double heterozygous for Hg S and C; FC: homozygous for Hg C; AF indeterminate variant: heterozygous for Hg A and indeterminate variant. *number of detected traits or hemoglobinopathies divided by all newborns tested in the period multiplied by 100.

**Figure 2:**
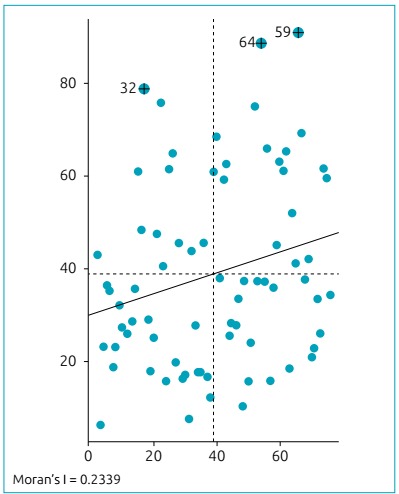
Moran dispersion diagram for the incidence of HbS and population of black and multiracial people in Sergipe, 2011-2012.

 HbAS presented a positive spatial correlation with the percentage of self-identified non-white individuals, indicating that, in Sergipe, both conditions have clustering characteristics (Moran’s Index 0*.*2339; p<0.001). According to the Moran scatter plot, the first quadrant of the coordinate system represented the spatial connectivity of the high observed value area unit surrounded by the high observed value region (High-high). The High-high clustering areas are mainly located in Aracaju (capital and main city of the state of Sergipe) and surrounding cities (Figure 2).

**Figure 3:**
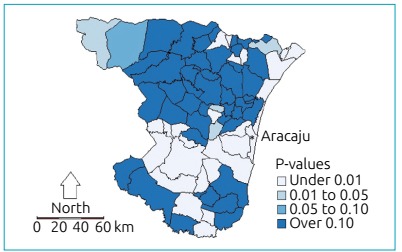
Spatial distribution by the LISA method testing the association between the incidence of HbS and the percentage of black and multiracial people in the cities of Sergipe, 2011-2012.

By using LISA, we detected hotspots of HbAS in the *Agreste* and Eastern regions, especially in Aracaju and surrounding cities (Figure 3).

**Figure 4:**
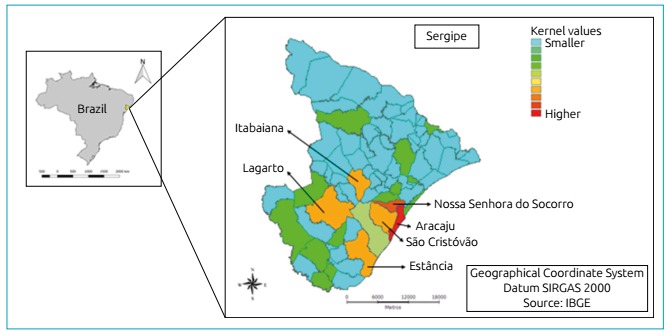
Kernel thematic map showing the density of SCT cases in the cities of Sergipe, 2011-2012.

Most cases of HbAS SCT were found in the cities of Aracaju (n=273; 22.7%), Nossa Senhora do Socorro (n=102; 8.4%), São Cristóvão (n=58; 4.8%), Itabaiana (n=39; 4.2%), Lagarto (n=37; 4.01%), and Estância (n=46; 4.9%) (Figure 4).

## DISCUSSION

The incidence of HbS has been detected by neonatal screening in several Brazilian states,^20^ ^,^ ^21^ ^,^ ^22^ and this study was the first to report it for Sergipe. Universal neonatal screening could identify affected babies before any symptoms, as well as asymptomatic heterozygous individuals, who can still transmit the gene to their offspring. The geographical distribution of this “silent” population is of extreme interest.

Sergipe still has *quilombola* communities, which are rural, suburban, or urban communities where enslaved descendants live and share a strong link to their African origins.^23^ *Quilombola* communities contributed to the maintenance of HbS areas because they used to be isolated and had many consanguineous marriages. They are still quite closed communities. The lack of miscegenation in these regions might have allowed the maintenance of the high incidence of HbS.

Geographical distribution has been used to study diseases in epidemiological analyses.^24^ ^,^ ^25^ ^,^ ^26^ ^,^ ^27^ This tool becomes important in the study of genetic diseases when there is a possibility of intervention with genetic guidance, as in the case of increased incidence of healthy carriers of the gene that causes the condition. Therefore, knowing the regions of Sergipe with higher incidence of heterozygous individuals is useful to guide the planning of health care actions for patients with SCA, as well as informing the situation to asymptomatic carriers and counseling the families, which may change the incidence profile of SCD in this population.

Screening for β-thalassemia trait in countries around the Mediterranean Sea region led to a drastic drop in the incidence of thalassemia cases because the affected families were informed about the carrier condition and had the opportunity to decide on their reproductive future.^28^ This experience raises the expectation that a similar approach with SCT individuals and their families could eventually affect the incidence of SCD. Regardless of the impact of screening in reducing the incidence of cases, individuals should be informed about their SCT condition to analyze their reproductive decisions better.

The distribution of new cases of HbS detected by the NSP is similar to that of cases of SCD in cities of Sergipe previously reported in another strategy.^29^ This result reinforces the need of support by health services in cities with a higher number of patients, focusing on treatment of acute events and medical follow-up, and informing asymptomatic heterozygotes and their families about the carrier condition.^30^

The incidence of SCT in Sergipe, according to NSP, was 2.7%, which is lower than the value estimated by Vivas^9^ in Aracaju (4.1%). This probably happened because the previous study estimated the HbAS proportion among blood donors, who may have agreed to a blood donation request from relatives with SCD.

The result of the universal screening in Sergipe reveals spatial randomness (p-value of black and multiracial people <0.05). We found a positive spatial correlation, that is, high values ​​of a variable will have high values of the same variable in their adjacency. The association between SCD and black individuals was present since the beginning of the disease characterization.^31^

In order to obtain the benefits of universal neonatal screening for hemoglobinopathies, besides the screening test, adequate medical follow-up should be available for patients with SCD, and their families should be informed about the condition.^32^ The implementation of these actions should be based on spatial distribution, prioritizing the regions with a higher incidence of HbS.

We emphasize that the data collection started in 2011, when the public health system implemented the universal neonatal screening in Sergipe. However, the population distribution changed since 2011 due to migrations, which can have modified the findings of this study. Also, we only assessed individuals treated in the public health system, so the frequency of hemoglobinopathies in the private system is not known.

Despite these limitations, we found a positive spatial correlation between the incidence of HbAS SCT and a large proportion of black and multiracial people, indicating a clustered characteristic of the condition in the state of Sergipe. We detected hotspots of HbAS SCT in the *Agreste* and Eastern regions, especially in Aracaju and surrounding cities.
